# Phosphatidic acid increases Notch signalling by affecting Sanpodo trafficking during *Drosophila* sensory organ development

**DOI:** 10.1038/s41598-020-78831-z

**Published:** 2020-12-10

**Authors:** Ignacio Medina-Yáñez, Gonzalo H. Olivares, Franco Vega-Macaya, Marek Mlodzik, Patricio Olguín

**Affiliations:** 1 Program of Human Genetics, ICBM, Facultad de Medicina, Universidad de Chile, Santiago, Chile; 2 Biomedical Neuroscience Institute, Department of Neuroscience, Facultad de Medicina, Universidad de Chile, Santiago, Chile; 3 Department of Cell, Developmental, & Regenerative Biology, Graduate School of Biomedial Sciences, Icahn School of Medicine at Mount Sinai, New York, NY USA; 4 Present Address: Millennium Institute for Integrative Biology (iBio), Department of Biology, Facultad de Química y Biología, Universidad de Santiago, Santiago, Chile

**Keywords:** Membrane trafficking, Cell biology, Cell signalling, Extracellular signalling molecules, Lipid signalling, Developmental biology, Differentiation, Genetics, Development, Genetic interaction

## Abstract

Organ cell diversity depends on binary cell-fate decisions mediated by the Notch signalling pathway during development and tissue homeostasis. A clear example is the series of binary cell-fate decisions that take place during asymmetric cell divisions that give rise to the sensory organs of *Drosophila melanogaster*. The regulated trafficking of Sanpodo, a transmembrane protein that potentiates receptor activity, plays a pivotal role in this process. Membrane lipids can regulate many signalling pathways by affecting receptor and ligand trafficking. It remains unknown, however, whether phosphatidic acid regulates Notch-mediated binary cell-fate decisions during asymmetric cell divisions, and what are the cellular mechanisms involved. Here we show that increased phosphatidic acid derived from Phospholipase D leads to defects in binary cell-fate decisions that are compatible with ectopic Notch activation in precursor cells, where it is normally inactive. Null mutants of *numb* or the *α-subunit of Adaptor Protein complex-2* enhance dominantly this phenotype while removing a copy of *Notch* or *sanpodo* suppresses it. In vivo analyses show that Sanpodo localization decreases at acidic compartments, associated with increased internalization of Notch. We propose that Phospholipase D-derived phosphatidic acid promotes ectopic Notch signalling by increasing receptor endocytosis and inhibiting Sanpodo trafficking towards acidic endosomes.

## Introduction

The Notch signalling pathway is an evolutionarily conserved cell–cell interaction pathway that plays a key role in boundary formation, lateral inhibition, and binary cell-fate decisions during the development of metazoans^1–3^. Notch signalling is tightly regulated by endocytosis and receptor trafficking after interaction with its ligands^4–6^. Moreover, besides interaction with its ligands, abnormal accumulation of Notch in endosomal compartments together with an acidic environment might lead to activation of the γ-secretase complex promoting ligand-independent activation of Notch signaling^7–11^.

In *Drosophila,* membrane lipids and lipid metabolism play an important role in modulating Notch signalling through regulation of membrane trafficking^12,13^. Loss of function of serine palmitoyltransferase and acetyl-CoA carboxylase results in the abnormal accumulation of Notch and other receptors in endosomal compartments generating tissue overgrowth phenotypes^14^. In addition, mutations in the gene coding for phosphatidylcholine cytidylyltransferase results in Notch loss of function defects in eye patterning and terminal photoreceptor morphology. These effects are associated with the accumulation of phosphatidylinositol (PI) and reduction of phosphatidylcholine (PC), two components of phosphatidic acid (PA) metabolism^15^.

**Figure 1:**
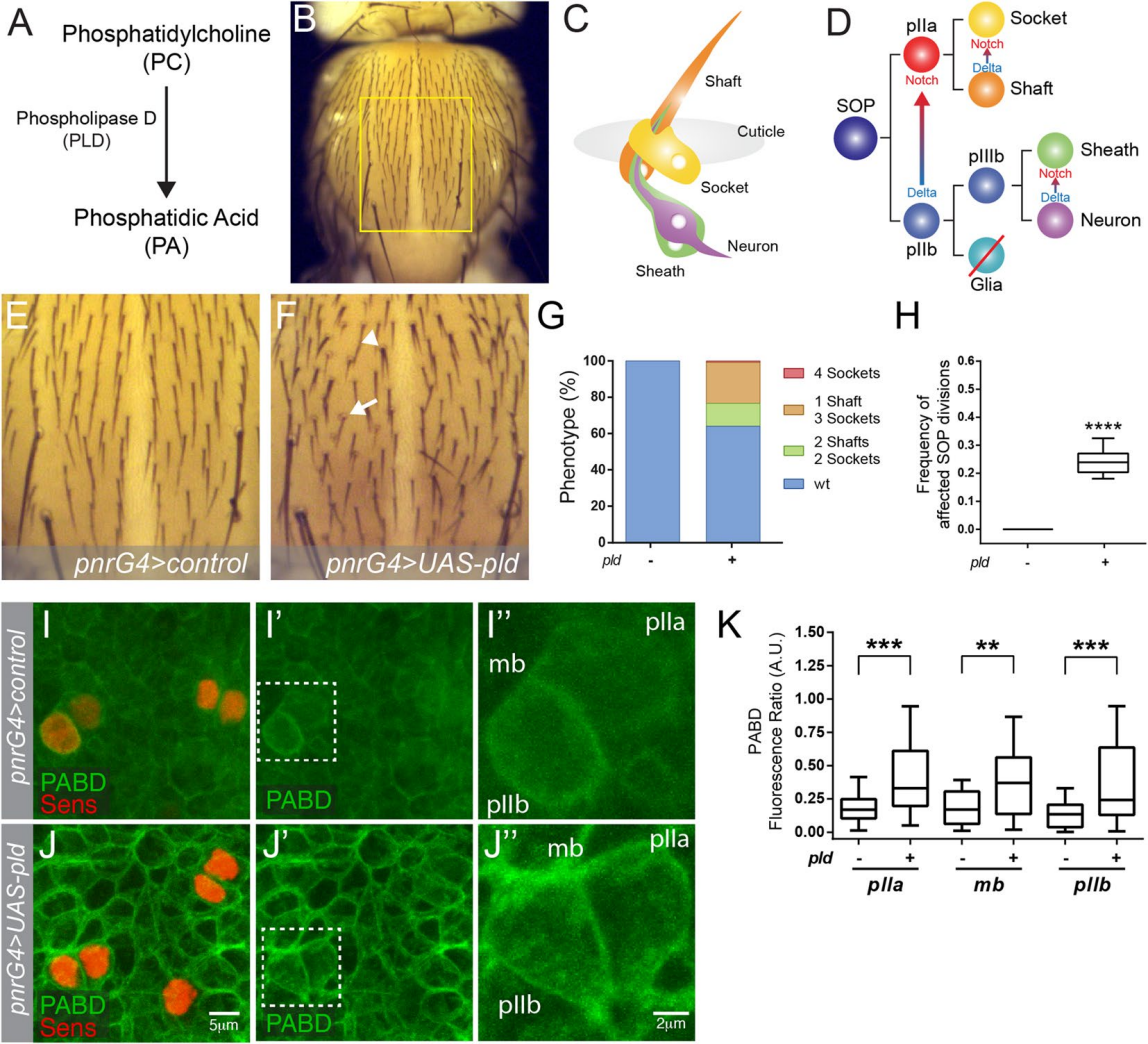
PLD-PA affects the cell-fates decisions during Sensory Organ development. (**A**) PLD uses PC as a substrate to generate PA. (**B**) Dorsal view of an adult fly thorax, yellow rectangle indicates the analyzed area. (**C**) The sensory organ (SO) is a mechanoreceptor composed of four cells. (**D**) Notch mediates binary cell-fate decisions of the SOP lineage. (**E**) Higher magnification of the analyzed area of a control fly. (**F**) Notum of a fly overexpressing PLD (PLD-GOF). The arrowhead indicates a SO with 2 shafts and 2 sockets. The arrow indicates a SO with 1sheath and 3socket. (**G**) Distribution of the 4 observable phenotypes in *pnrG4* > *pld* notum. (**H**) Quantification of the frequency of divisions affected by the gain function of Notch (n = 10, p < 0.0001). (**I**,**J″**) Confocal projection of notum epithelium stained with senseless (red) and PABD-GFP (green). (**I″**,**J″**) Magnification of a dividing SOP, specifying each cell and the midbody. (**K**) Quantification of fluorescence ratio in both conditions (n = 23, **p < 0.01, ***p < 0.001). The scale represents 5 µm (**I**–**I′**,**J**–**J″**) and 2 µm (**I″**,**J″**).

PA is mainly synthesized by two enzyme families: Diacylglycerol Kinase (DGK) and Phospholipase D (PLD)^16^. PLD catalyses the hydrolysis of PC to generate PA (Fig. 1A), which is present in almost every cell membrane^17^. The role of PA derivatives in regulating signalling is not limited to the Notch pathway. The role of phospholipase D-derived PA (PLD-PA) in promoting EGFR endocytosis and inhibiting its lysosomal degradation is well known^18,19^. However, whether Notch signalling is affected by PLD-PA levels is unknown.

The development of the mechanosensory bristles of *Drosophila*, known as sensory organs (SO), is one of the best systems for the study of Notch signalling. The SO comprises four cells: the shaft, the socket, the sheath, and the sensory neuron (Fig. 1B,C). These originate from a single precursor cell called the SO precursor (SOP) or pI cell, which undergoes successive asymmetric cell divisions (ACDs) (Fig. 1D). During this process, the cell-fate determinant Numb segregates unequally in one of the daughter cells inhibiting receptor signalling by preventing the recycling of Sanpodo (Spdo)^20–23^, which interacts with Notch and is required for both signalling activation in pIIa daughter cell and inactivation in pIIb^22,24,25^. Spdo is a four-pass transmembrane protein, expressed in asymmetrically dividing cell lineages^24,26–30^. In SOP linage, unlike cell-fate determinants, Spdo is distributed to both pIIa and pIIb cells after mitosis, and its localization is primarily at the plasma membrane^22,28,31^. Spdo's primary role occurs in the pIIa cell, promoting Notch activation by directing receptor trafficking and binding to γ-secretase^25^. Altered function of Spdo in SOP results in bristle loss, a Notch loss of function phenotype^31–33^. Conversely, abnormal upregulation of Notch during ACDs results in altered SO phenotypes, including SO composed by double shafts or multiple sockets^34,35^.

Here we show that increased levels of PLD-PA in the notum alters cell-fate decisions during SOP differentiation favouring shaft and socket cell-fates. Molecular and epistatic analysis suggests that this phenotype is generated by the accumulation of PA and no other PA metabolic fate, such as the phosphoinositides. Genetic interactions suggest that this effect is due to Notch gain of function. In vivo analyses show that high PLD-PA results in increased endocytosis of Notch and a diminished sorting of Spdo towards late endosomes. Accordingly, increased PLD-PA results in enlarged early endosome compartment marked by Rab5. Together, these data suggest that PLD-PA can regulate the binary fate decision by activating Notch signalling through the modulation of Notch and Spdo endosomal trafficking during organogenesis.

## Materials and methods

### Fly strains and genetics

Overexpression studies were performed using the Gal4/UAS system^36^. We used the following UAS lines: UAS-Dl, UAS-Ser, UAS-mCD8-ChRFP (Bloomington *Drosophila* Stock Center; BDSC), UAS-*fwdIR, UAS*-*sktlIR,* UAS-*plc-21IR,* UAS*-dgkIR,* UAS-*gfpIR* (Vienna *Drosophila* RNAi Center). *UAS-pld38,* and *UAS-rdgA/dgk* were a generous gift from Padinjat Raghu (National Centre for Biological Sciences, Tata Institute of Fundamental Research, India). We also used the following Gal4 lines: *pnr-Gal4, en-Gal4* and *sca-Gal4* (BDSC). We also used the following strains *Dl*^*[Rev10]*^, *Ser*^*[RX82]*^, *Spdo*^*G104*^, *Ap-2α*^*[40–31]*^, *numb*^*15*^^37^. *SpdoCh2GFP3, neur*-nlsFP670, *NiGFP* were a generous gift from François Schweisguth (Institut Pasteur, France). *UAS-lipinwt* was a generous gift from Michael Lehmann (University of Arkansas, USA). All phenotypes were analysed at 25 °C unless stated otherwise.

### Quantification of adult phenotypes

To quantitate cell-fate defects in the SO caused by genetic manipulations, a correction was made based on the number of cells affected by the Notch gain of function. To have a normalized value for each phenotype, it was considered that “the frequency of affected SOP ($f$)″ is the number of times a phenotype (2shafts, 2sockets; 1shaft, 3sockets; and 4sockets) is repeated, multiplied by the number of cell divisions affected in that phenotype, divided by the total number of mechanoreceptors (*Positions*) multiplied by the 3 cell divisions that each sensory organ undergoes:

$$f=\frac{\left(2shafts, 2sockets \; x1\right) +\left(1shaft,3sockets \; x 2\right) +(4sockets \;x 3) }{Total \; Positions \; x3}$$

Higher frequencies score indicates that more cell divisions undergo a double-Notch signal activation, i.e., in a 4-socket phenotype, both cells in all 3 divisions acquired a Notch+ cell-fate (Figure S1H–H″).

### Immunofluorescence and antibodies

Pupae were aged for 16 to 18 hours after puparium formation (hAPF) for SOPs to reach the two-cell stage, dissected in 1× phosphate-buffered saline (1× PBS) and then fixed for 20 min in 4% paraformaldehyde (PFA) at room temperature. Dissections were as previously described in Jauffred and Bellaiche^38^. Primary antibodies used were: mouse anti-Notch extracellular domain (NECD, C458.2H, 1:100; Developmental Studies Hybridoma Bank; DSHB), rat anti-Elav (7E10, DSHB, 1:50), mouse anti-Prospero (MR1A,DSHB, 1:10), rat anti-DE-Cad (DCAD2, DSHB, 1:100), rabbit anti-GFP (Thermo Fisher, 1:1000), guinea pig anti-Senseless (1:1000)^39^ a generous gift from Hugo Bellen (HHMI, Baylor College of Medicine). FITC, TRITC, Alexa Fluor 647-coupled secondary antibodies (1:100) were from Jackson’s Laboratories.

### Phosphatidic acid probe

PABD1x-GFP probe was kindly provided by Guangwei Du from University of Texas Health Science Center at Houston, Houston, TX, USA. The probe was amplified by PCR and inserted in pCaSpeR-act (DGRC, stock number:1067)^40^ using *KpnI* and *NotI*. The injection of pCaSper-act-PABD1x-GFP construct was performed by BestGene (Chino Hills, California. E.E.U.U.).

### Imaging

Fixed nota images and live imaging were acquired on a confocal microscope Olympus IX81. All images were processed and assembled using ImageJ 1.48 and Adobe Illustrator 2020.

As for the fly thoraxes, adult females were selected and fixed for 24 h in 70% ethanol. The samples were placed on Sylgard plates with 70% ethanol, legs and wings were removed avoiding damage. After immobilizing the sample, the ILUMINA software was used to take a picture of the thoraxes at 6× with a stereoscopic magnifier attached to an INFINITY Lumenal Photo Camera 1.

Fluorescence quantifications ($fr$) were performed using ImageJ software. The method described by Bohdanowicz et al. was used as a reference for the fluorescence measurement of the phosphatidic acid sensor^41^. This method consists in the measurement of the plasma membrane fluorescence ratio normalized by the cytoplasmic fluorescence: $fr= \frac{Plasma \;Membrane-Cytoplasm}{Cytoplasm}$

For nuclear GFP, *NiGFP* was measured in small z-stacks centred at the nuclear level. We used the same principle described above, but in this case, we normalize with GFP of an epithelial cell. Nuclei were identified using the SOP-specific Senseless marker. ROIs delimiting the nuclei were drawn manually. $fr= \frac{SOP\; cell - Epithelial \; cell}{Epithelial \; cell}$

For in vivo imaging, Spdo punctae were manually counted in each plla and pllb cells Additionally, every punctae volume (V) was calculated, assuming an ellipsoid shape: $V= \frac{4}{3}\pi a{b}^{2}$

For this calculation both punctae axes (*a*, the longest and *b*, the shortest) were manually measured.

### Notch endocytosis assay

To label the internalized fraction of Notch, *pnr-Gal4* and *pnr-Gal4*>*uas-pld38* nota explants at 15 hAPF were first incubated with the anti-NECD antibodies, that recognize the extracellular portion of Notch receptor, for 10 min (pulse). Following the pulse, medium containing anti-NECD antibodies was washed three times with 1× PBS and nota were either fixed with 4% PFA (i.e. initial internalized Notch fraction) or kept for 15 min (chase) at 25 °C to allow endocytic traffic of NECD (i.e. endocytic and post endocytic Notch fraction) and subsequently fixed in 4% PFA and immunostained as described above.

Internalized anti-NECD antibodies were quantified by counting punctae along the apical-basal axis. The cell was divided into three apical-basal regions: (sub)Apical, Mid, and Basal. E-cadherin staining was used as a reference.

### Statistical analysis

Statistical analysis was carried out using Dunnett’s multiple comparisons for adult phenotype quantifications and two-tail Student’s t-test for the immunohistochemistry experiments. Specifically, for the distribution analysis of Rabs along the apical-basal axis, we used a Tukey's multiple comparisons. Statistical significances are represented as follows: *p < 0.05, **p < 0.01. ***P < 0.001, ****p < 0.0001.

## Results

### PLD-PA affects binary cell-fate decision mediated by Notch during the development of the sensory organ

We first asked whether increased PLD-PA in *Drosophila*^42^, influences binary cell-fate decisions mediated by Notch during the development of the SO (Fig. 1). Overexpression of *Drosophila* PLD in the central region of the notum, driven by *pnr-Gal4* driver, resulted in an altered cellular composition of the SOs, including organs comprising 2 shafts and 2 sockets, or 1 shaft 3 sockets (Fig. 1E–G). In addition, we found an increase of bristles upon PLD overexpression (Figure S1A). To confirm that altered cellular composition of the SOs are due to alterations of cell-fate decisions, we analysed the expression of molecular cell-fate specific markers at 24 hours after puparium formation (hAPF) (Figure S1B–D). We found clusters with two cells expressing Prospero (Pros), a marker of glial sheath cells^43^, indicating that fate acquisition after pI division occurred normally, generating pIIb and pIIa, however, pIIb generates two sheaths (Figure S1E,E′). We also found clusters that only express the SOP lineage marker Senseless (Sens)^39^, but not Elav, a neuronal marker (Figure S1B,C)^44^, or Pros, indicating that pI cell gave rise to a pIIa-like cell instead of pIIb, which generates two external cells. This phenotype could correspond to one of the 3 different phenotypes observed in SOs of the adult thorax: 2 sheath-2 sockets (Figure S1F–F″), 1 sheath-3 sockets (Figure S1G–G″) or 4 sockets (Figure S1H–H″). To evaluate quantitatively the phenotype, we considered the number of adult visible cell-fates affected per cell division over the total of cell divisions analysed (Figure S1F–H″). Note that although this methodology underestimates the effect on binary fate decisions of cells, since it does not consider some fate decisions of pIIa daughter cells, it allows quantitative comparisons between different genetic conditions. The frequency of binary cell-fate decisions affected is around 0.25 in nota overexpressing PLD under the control of *pnrGal4*, compared with the absence of alterations in flies that only carry the *pnrGal4* driver (Fig. 1H). The low penetrance could be due to compensatory activity of other enzymes within the PA metabolic pathway. To confirm that this result is not due to an unspecific interaction between the *UAS-PLD* and the *pnrGal4* insertions, we used the *scaGal4* driver, which is only expressed in the proneural clusters (Figure S2A–D)^45^. This genetic condition results in similar cell-fate defects, although weaker than with *pnrGal4* (Figure S2E,F).

To answer whether altered cell-fate decisions are due to enhanced Notch signalling, we performed genetic interaction analyses. Removing one copy of Notch partially suppresses the phenotype associated with PLD overexpression (Figure S3). Interestingly, removing one copy of both Notch ligands, Delta and Serrate, enhances the effects of PLD overexpression, consistent with their role as inhibitors of Notch signalling in the same cell (cis-inhibition) (Figure S3)^46^. Thus, these results strongly suggest that the phenotype caused by PLD gain of function is mediated by the overactivation of Notch signalling in cells where the signal should be inactive (e.g. pIIb). To confirm this, we expressed the Notch Intracellular Domain (NICD) fused with GFP as a readout of signaling activity (Figure S4)^47^. We observe an increase of NICD fluorescence in the PLD overexpression background, as compared to the control (Figure S4A–C). Moreover, we found that the signal asymmetry between plla and pllb, as seen in control SOP cells, is lost after PLD overexpression (Figure S4D).

Moreover, we did not observe alterations in wing patterning in flies expressing *UAS-pld* under the control of the *engrailed-Gal4* driver, suggesting that PLD overexpression only affects cells that undergo ACD (Figure S5).

Finally, to confirm that PLD overexpression affects PA levels in epithelial cells of the notum, we expressed a PA sensor that comprises the PA binding domain of Spo20p fused to EGFP (PABD-GFP) (Fig. 1I–K)^41^. Overexpression of PLD in the notum epithelium results in a significant enrichment of the sensor signal at the plasma membrane compared with the cytoplasm signal (Fig. 1I–K).

Together these results led us to propose that the effects of PLD overexpression in cell-fate during SOP development are due to increased Notch signalling.

### Binary cell-fate decisions of the SOP are affected by the accumulation of PLD-PA

**Figure 2:**
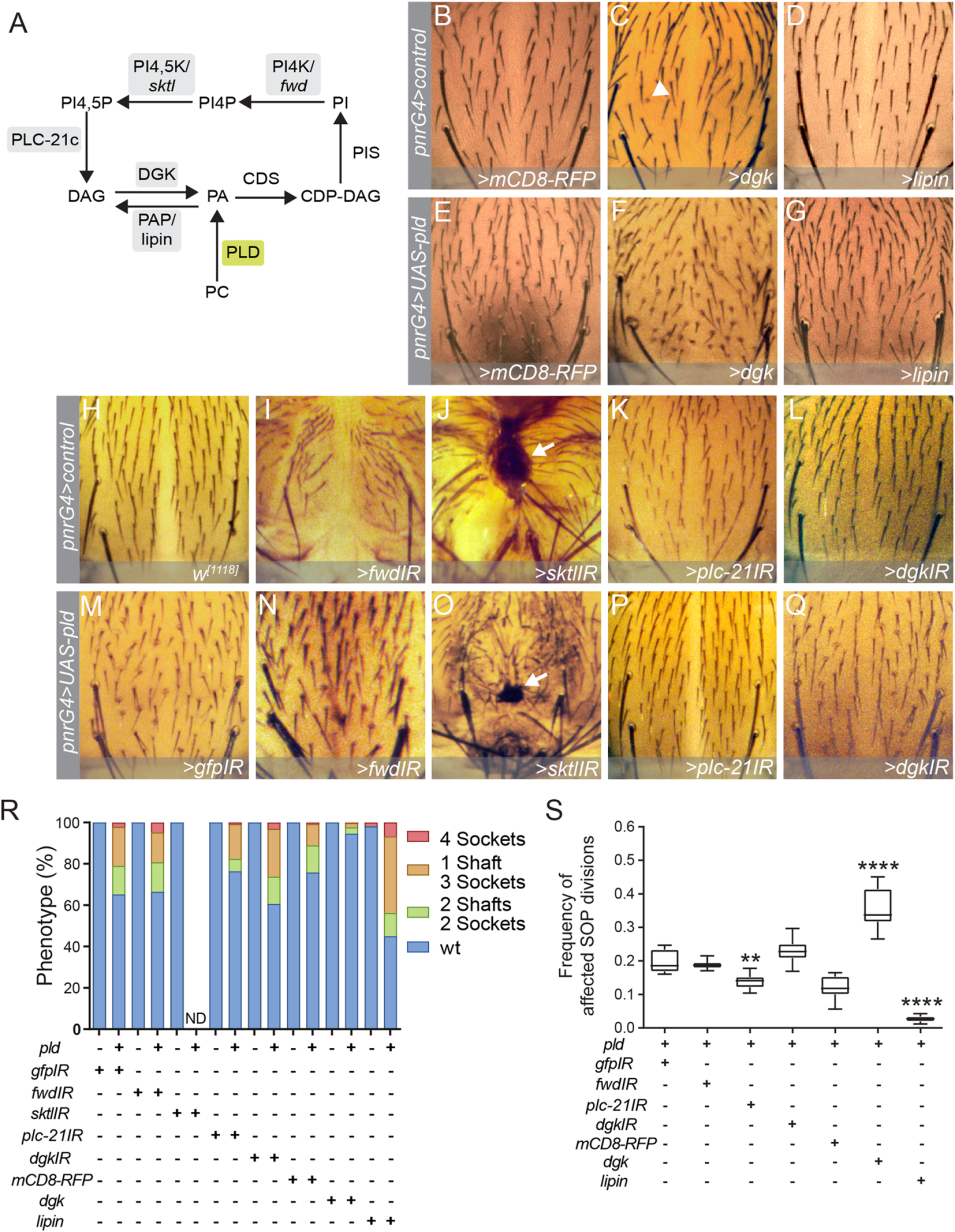
PLD-PA associated cell-fate defects are due to PA and not to PIPs or DAG accumulation. (**A**) Metabolic fates of PA. Gray boxes indicate the upregulated or downregulated enzyme. Green box indicates PLD. (**B**–**G**) Dorsal views of nota expressing UAS constructs targeting enzymes that control PA metabolism. Note that the overexpression of DGK in the central region of the notum directed by *pnrGal4 (pnr* > *dgk*) results in the phenotype of 4 sockets, indicated by an arrowhead, in ~ 2% of the sensory organs analyzed, with 50% penetrance. (**H**–**Q**) Dorsal views of nota expressing UAS-RNAi (IR) constructs targeting enzymes that control PIPs and DAG metabolism. Note that the interference of the expression of these enzymes results in serious defects of the notum morphology, *pnrG4* > *fwdIR* (**I**) *pnrG4* > *sktlIR* (**J**) *and pnrG4* > *pld,sktlIR* (**O**). The black scars associated with defective heminota fusion or migration (**J**,**O**) are indicated by arrows. Interestingly, these defects caused by reduced expression of *fwd* and *sktl* in the pupae, are partially rescued by the overexpression of PLD. (**R**) Distribution of the 4 phenotypes for each case. (**S**) Quantification of the frequency of SOP divisions affected by the gain function of Notch (n = 10, ND = not determined **p < 0.01, ****p < 0.0001).

To answer whether the phenotype associated with PLD overexpression is mediated by PA accumulation, we reasoned that increasing or decreasing PA levels genetically will result in an enhancement or suppression of the phenotype, respectively. Overexpression of the enzyme Diacylglycerol kinase (DGK)^42^, which catalyses the synthesis of PA from DAG (Fig. 2A), enhances the phenotype associated with PLD overexpression (Fig. 2C,F,R,S). Accordingly, Lipin overexpression, an intracellular phosphatidate phosphatase that converts PA back to DAG (Fig. 2A), supresses almost completely the PLD gain of function phenotype (Fig. 2D,G,R,S)^48^. These data strongly argue that accumulation PA is responsible for the Notch gain of function phenotype.

Downstream on the PA metabolic pathway is the phosphoinositides (PIPs) synthesis (Fig. 2A). Since some of these metabolites have been directly related to the regulation of Notch signalling^49–51^, we asked whether the phenotype associated with the increase of PLD-PA could be generated by an accumulation of PI4P, PI(4,5)P2 or DAG (Fig. 2H–Q).

To generate a genetic condition that would result in the accumulation of PA but not of PIPs, we reduced the expression of the kinases PI4K/*fwd* (Fig. 2I,N) or PI4,5K/*sktl* (Fig. 2J,O) in animals that overexpress PLD. Knockdown of these genes by using specific double-stranded interference RNAs (IR), resulted in morphological defects reminiscent of defective heminota fusion or migration towards the midline^52,53^. Nevertheless, PI4K/*fwd* knockdown does not modify the phenotype associated with high levels of PLD-PA (*p* = 0.9925) (Fig. 2S), suggesting that accumulation of PI4P or PI(4,5)P2 is not responsible for the Notch gain of function phenotype. Although it was not possible to perform quantitative analysis in animals expressing a *sktl IR*, since its strong effect in the notum morphology (Fig. 2O), the defects associated with the gain of PLD function are still detected. Interestingly, the visible defects on the morphology of the notum are partially rescued by PLD overexpression (Fig. 2N,O). These data indicate that PLD-PA effects on binary cell-fate decisions are not due to the accumulation of phosphoinositides.

Finally, we evaluated the potential role of DAG accumulation (Fig. 2K,P). Directed expression of an RNAi against *PLC-21c* does not affect cell-fate of the SOP lineage (Fig. 2K). However, it decreases the frequency of phenotypes associated with overexpression of PLD in SO and partially rescues its expressiveness (*p* = 0.0062) (Fig. 2P,R,S). This suggest that the accumulation of DAG might be in part responsible for the observed phenotype. Nevertheless, decrease in *dgk* expression does not generate a visible phenotype on its own (Fig. 2L,Q) and does not enhance the phenotype caused by the gain of PLD function (Fig. 2R,S). In contrast, its gain of function enhances the phenotype associated with PLD overexpression (Fig. 2F,S).

Together, these results suggest that the accumulation of PLD-PA, and not of PIPs or DAG, is responsible for cell-fate defects of the SO.

### PLD-PA effects on binary cell-fate decisions are suppressed by Spdo loss of function mutants and enhanced by Numb and AP-2α

**Figure 3:**
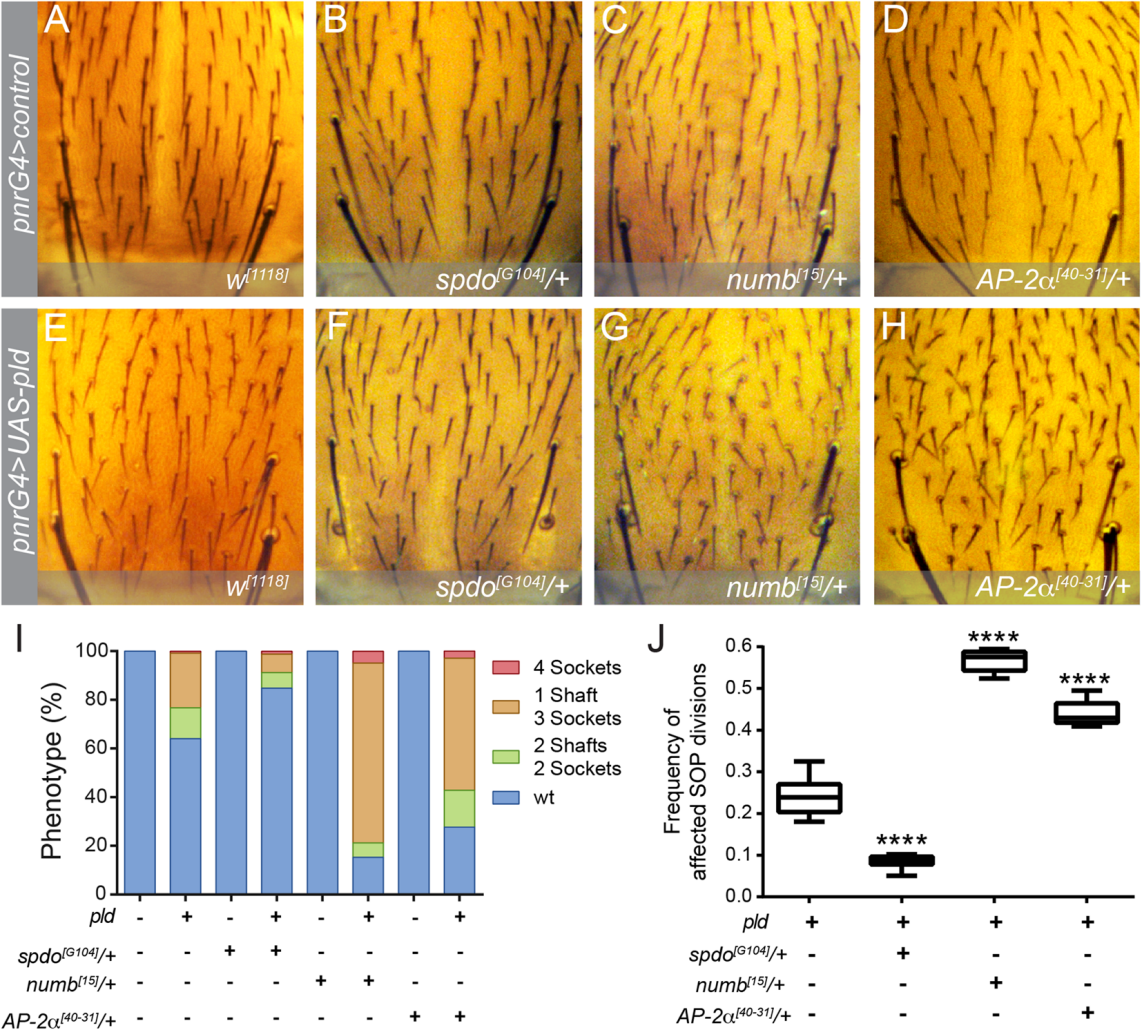
Notch trafficking regulators modify the PLD overexpression phenotype. (**A**–**H**) Dorsal views of nota on a heterozygous background of different Notch regulators that control the cell-fate in pllb. (**A**–**D**) Driver control. (**E**–**H**) Overexpression of PLD. Note that in heterozygous backgrounds there are no changes in the SO cell-fates. (**I**) Frequency of the 4 phenotypes for each case. (**J**) Quantification of the frequency of SOP divisions affected by the gain function of Notch. n = 10, ****p < 0.0001.

Since Numb and Spdo regulate Notch signalling in pIIa and pIIb by controlling receptor trafficking^20–22,25^, we asked whether reducing their genetic dose by half modifies PLD gain of function phenotype (Fig. 3). Heterozygous flies for *spdo*^*G104*^ and *numb*^*15*^ alleles show normal SOs (Fig. 3B,C,I). However, *spdo*^*G104*^/ +  diminishes the frequency of 1shaft/3sockets and 2 shafts/2sockets phenotypes and the frequency of affected SOP lineage ACDs (p < 0.0001) (Fig. 3F,I,J). In contrast, *numb*^*15*^/+ enhances the frequency of 1shaft/3sockets and the total frequency of affected ACDs (Fig. 3G,I,J). These data strongly suggest that the PLD-PA phenotype is directly related to Notch signalling through the regulation of Spdo and Numb in the pllb cell.

Cell-fate specification during SOP development depends on the endosomal localization of Spdo and Notch, which requires adaptor protein complex 2 (AP-2)^20,30^. Since overexpression of PLD leads to membrane transport defects in photoreceptor cells^54^, we asked whether reducing the genetic dose of AP-2α modifies PLD-PA associated phenotypes (Fig. 3). Heterozygous animals for the null allele, AP-2α^[40–31]^, do not affect cell-fate decisions during SOP development (Fig. 3D). Strikingly, it strongly enhances the frequency of PLD-PA associated phenotypes and the total frequency of affected SOP divisions (Fig. 3H–J). These results suggest that overexpression of PLD could be affecting trafficking of the Spdo-Notch complex in the pllb cell.

### PLD-PA promotes Notch endocytosis and inhibits Spdo trafficking towards acidic compartments

Since PLD-PA induces the endocytosis of EGFR and inhibits its lysosomal degradation^18,19^, we asked whether it may affect Spdo-Notch complex endocytosis and trafficking toward late acidic endosomes.

**Figure 4:**
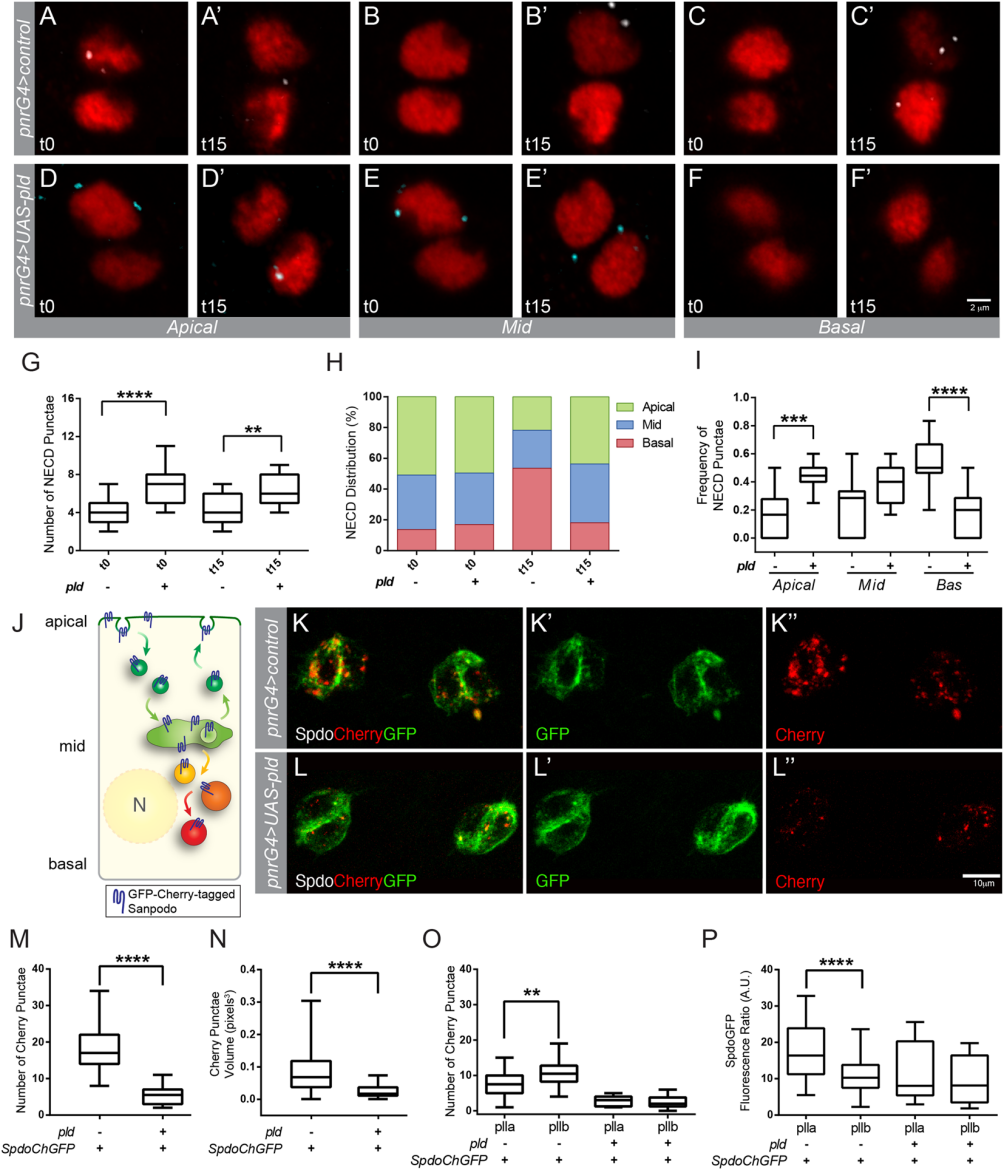
PLD-PA increases endocytosis of Notch receptor and decreases Spdo sorting towards late endosomes. (**A**–**I**) Pulse and chase in apical, middle, and basal planes using anti-NECD (cyan) to mark the Notch receptor and anti-senseless (red) to identify the SOP cells. (**A**–**F**′) Confocal projections of pIIa (upper) and pIIb (bottom) in 16 hAPF pupae, in control conditions (**A**–**C**′), and overexpression of PLD (**D**–**F**′). Note that in the mid and basal planes there are no NECD+ punctae at t0 but they appear at t15. In contrast, in a PLD overexpression background, NECD is present in the mid plane in both t0 and t15 but there is no signal in the basal plane. (**G**) Quantification of the total NECD+ punctae. (**H**) Spatial distribution of NECD + punctae across the cell. (**I**) Quantification of NECD positive dots frequency at t15. (**J**–**O**) In vivo imaging of Sanpodo double-labeled with Cherry/GFP during asymmetric SOP division. (**J**) Schematic representation of *SpdoCherryGFP* distribution along the apico-basal axis. GFP fluorescence (green) is detected at the membrane, early and recycling endosomes. Conversely, Cherry fluorescence (red) is detected in late endosomes and lysosomes. This difference in the punctae fluorescent signal is due to late endosomes and lysosomes have an acidic pH that turns off GFP fluorescence, while the Cherry molecule has a slower maturation time than GFP, so it is not initially detected during endocytosis and protein recycling^47^. (**K**–**K″**) Spdo in vivo imaging in pIIa (left) and pIIb (right) in pupa control. (**L**–**L″**) Spdo in vivo imaging in pupae with overexpression of PLD. (**M**) Quantification of the total Cherry positive dots. (**N**) Volume quantification of Cherry dots. (**O**) Quantification of Cherry positive dots in plla and pllb. (**P**) Quantification of GFP fluorescence in plla and pllb. ((**D**–**F′**; t0 n = 16, t15 n = 11 **A**–**C′**; t0 n = 26, t15 n = 13; **K**–**L″** n = 20 **p < 0.01, p*** < 0.001, ****p < 0.0001). The scale represents 2 µm (**A**–**F′**) and 10 µm (**K**–**L″**).

First, we perform an *ex-vivo* endocytosis assay in the notum epithelium to answer whether PLD-PA affects Notch trafficking (Fig. 4A–F′)^55^. In brief, the dorsal part of the nota was dissected 15 h after puparium formation and cultured in the presence of anti-Notch antibodies that recognize the extracellular domain (NECD). After washing the antibody, the internalization of the antibody-bound receptor was detected at 0 min (t0) and 15 min (t15). First, we observed a significant increase in the number of internalized anti-Notch antibodies marks at t0 and t15 upon PLD overexpression, suggesting that PLD-PA promotes receptor endocytosis (t0 p < 0.0001, t15 p = 0.0080) (Fig. 4G). Then, we studied the distribution of internalized anti-Notch antibodies signal along the apical-basal axis (Fig. 4H). At t0 PLD-PA does not affect Notch distribution along the apical-basal axis. However, at t15 PLD-PA affects the distribution of internalized Notch, which remains towards the apical portion of the pIIa and pIIb cells (Fig. 4H). The increase in PLD-PA results in 2.5 times more receptor signal at the apical portion of the cells (*p* = 0.0001, Fig. 4A,A′,D,D′,I). No significant differences were found in the mid-region of the cell (*p* = 0.0698, Fig. 4B,B′,E,E′,I). At the basal level, the increase in PLD-PA results in a threefold decrease in receptor signal (*p* < 0.0001, Fig. 4C,C′,F,F′,I). This apical accumulation could be explained by an increased transport of basal Notch to apical endosomal compartments, a decreased transport from early to late endosomes and/or higher basal receptor degradation.

To answer whether changes in receptor localization are associated with changes in early and late endosomal compartments, we analysed the distribution, number and volume of puncta marked with Rab5 and Rab7, respectively (Figure S6A–D′). We did not observe changes in their distribution along the apical basal axis, (Figure S6E,F). However, we detected an enlargement of the mean volume of Rab5+ puncta and a slight decrease in the volume of Rab7+ puncta (Figure S6G). These data support the notion that accumulation of Notch at apical endosomes could be due to effects on the size of early and late endosomal compartments.

Since Numb inhibits Notch signalling in pIIb by directing Notch and Spdo to acidic endosomes and degradation^47,56^, we evaluated whether Spdo is sorted towards late endosomes in animals expressing high levels of PLD-PA. We used a Spdo protein double-tagged with GFP and Cherry, which allows us to distinguish its localization between early and late acidic endosomes^47^. When Spdo-GFP/Cherry is localized to the plasma membrane, in early or recycling endosomes, GFP signal predominates, whereas Cherry signal predominates when localized to late endosomes and lysosomes (Fig. 4J)^47^. A high GFP/low Cherry ratio is found throughout the cell membrane and in early/sorting endosomes, whereas low GFP/high Cherry ratio is found in more acidic endosomes (Fig. 4K–K″)^47^. PLD overexpression (Fig. 4L–L″) does not yield any qualitative change in GFP mark (Fig. 4L′), however, the Cherry punctae decreases in number and size (Fig. 4L″). The number and volume of Cherry punctae in plla and pllb decreases 3.5- and 4 times, respectively, in nota with high PLD-PA levels (*p* < 0.0001, Fig. 4M,N). Strikingly, the difference in the number of Cherry punctae between pIIa and pIIb that is observed in the control condition (p = 0.0083, Fig. 4O), is not found in animals that overexpress PLD (p = 0.7522, Fig. 4O). A similar lack of asymmetry is also observed when comparing GFP fluorescence in control condition (*p* = 0.0092, Fig. 4P) and PLD overexpression (*p* = 0.4033, Fig. 4P). These results suggest that the trafficking of the Notch-Spdo complex towards late endosomes is disrupted by PLD-PA.

## Discussion

Here we show that PLD-PA affects binary cell-fate decisions during SO development by increasing Notch signalling. This observation is supported by the potentiation of the PLD overexpression phenotype by overexpression of DGK. Interestingly, the effects of DGK overexpression are much weaker than the effects associated with the overexpression of PLD, suggesting the PA generated by PLD is more active or abundant than PA generated by DGK from DAG. Accordingly, higher PC content in epithelial cells compared to that of DAG could explain this difference^57^.

Epistatic and image analyses using the PABD-GFP PA sensor^58^ suggest that high levels of PLD-PA, and not phosphoinositides, results in enhanced Notch function. Unexpectedly, we found that *PLC-21c* knockdown suppresses PLD-PA associated cell-fate defects. We suggest that this might be due to the accumulation of PI(4,5)P2, a molecule that in vitro can prevent the association of the γ-secretase with its substrates, thus inhibiting Notch signalling^59^. More studies will be necessary to confirm that inhibition of γ-secretase activity mediated by PI(4,5)P2 accumulation occurs in vivo. To understand more precisely the role of phospholipids in binary cell-fate decisions of the SOP lineage, it will be necessary to investigate the composition of the lipid membrane of SOP during differentiation through lipidomics using shotgun Mass Spectroscopy (MS) methods^60–62^.

In *Drosophila* photoreceptor cells, PLD-PA regulates membrane trafficking, mainly by promoting the internalization of receptors and favouring recycling at the expense of trafficking to acidic endosomes and lysosomes^54^. In mammalian cells, PLD-PA promotes ligand-independent internalization of EGFR and inhibits its lysosomal and proteasomal degradation^18,19^. This effect on EGFR could explain the increase of SO numbers observed, since positive feedback between the phenotype and the pathway has already been described^63,64^. Since vesicular trafficking plays a fundamental role in the regulation of Notch signalling and hence also in the correct development of the SO^65,66^, we proposed that PLD-PA modulates endosomal trafficking of Notch and could enhance its signaling in a ligand-independent way. Two pieces of evidence support this hypothesis of ligand-independent signaling in our model. First, we observed here that removing a copy of the ligands Delta and Serrate increases the Notch gain of function phenotype. Coincidently, it has been reported that cis-interactions between Notch and its ligands inhibit Notch ligand-independent signalling during *Drosophila* oogenesis^67^. Second, the Rab5+ vesicles enlargement is associated with defects in the *lgd* gene, which is also involved in Notch ligand-independent activation^49,68–71^. Also, in human cell culture assays, ESCRT-III has been shown to interact with MITD1, which has a similar structure with proteins of the PLD superfamily^72^. Since the loss of ESCRT function leads to ectopic activation of Notch signaling in a ligand-independent manner, the interaction between PLD-PA and this complex in this model should be investigated^9,73–77^.

Several pieces of evidence support that PLD-PA modulates endosomal trafficking: first, we found that mutants of *numb* and *spdo*, two regulators of Notch trafficking and signalling during ACDs^20,24^, enhance or suppress dominantly the cell-fate defects generated by PLD-PA, respectively; second, PLD-PA promotes the internalization of the receptor; third, it results in the enlargment of early endosome compartment Rab5+ ; and fourth, PLD-PA precludes trafficking of Spdo towards acidic endosomes. Thus, increasing the levels of PLD-PA made Spdo available in both SOP daughter cells for activating Notch signalling symmetrically. We hypothesize that the observed activation mechanism is similar to Notch ligand-independent signaling. However, in contrast to this mechanism, the activation does not appear to occur in acidic compartments, and associated with the accumulation of Spdo and Notch in early endosomes. This association could explain the absence of a phenotype in wing tissues where *spdo* is not expressed^24^.

Unexpectedly, we found that halving the genetic dose of AP-2 strongly enhances the PLD-PA effect. This can be explained by recent studies that shows that AP-2 regulates autophagosome turnover independent of its role in endocytosis^78^. Moreover, in neurons lacking AP-2 the trafficking of the β-secretase (BACE-1) towards the lysosomes is diminished and results in an increase of the activity at the plasma membrane^79^. Similarly, Numb has been identified as a new mediator of the autophagic process. Numb knockdown results in changes in lysosomal acidic environment and decreased activity of glycosylated LAMPs and Rab7, leading to the impairment of autophagic degradation by inhibiting the activation of lysosomal enzymes^80^.

To our knowledge, this is the first report that links PLD-PA to the regulation of the Notch signalling pathway. We propose that this function has been ignored since it depends on Spdo activity, which regulates Notch signalling only in cells that undergo ACDs^24^.

Future work will be required to determine whether PLD-PA regulates Notch signalling in human cells that undergo ACDs, such as stem cells of the gastric antrum^81^. These cells undergo symmetric divisions during tumorigenesis, therefore, to study the role of PLD-PA in these cells may contribute to the development of therapeutic strategies to prevent tumour progression.

## Supplementary Information

Supplementary Information.

## Data Availability

The datasets generated for this study are available on request to the corresponding author.
