# Supplementary material for: Phosphatidic acid increases Notch signalling by affecting Sanpodo trafficking during Drosophila sensory organ development
Source: Sci Rep. 2020 Dec 10;10:21731. doi: 10.1038/s41598-020-78831-z (PMC7729928; doi:10.1038/s41598-020-78831-z)
Supplement: Supplementary file 1 — Supplementary Information. [file 41598_2020_78831_MOESM1_ESM.pdf]

Supplementary Information

Phosphatidic acid increases Notch signalling by affecting Sanpodo trafficking during *Drosophila* sensory organ development

Ignacio Medina-Yáñez[1,2], Gonzalo H. Olivares[1,2], Franco Vega-Macaya[1,2 †], Marek Mlodzik[3], Patricio Olguín[1,2]*

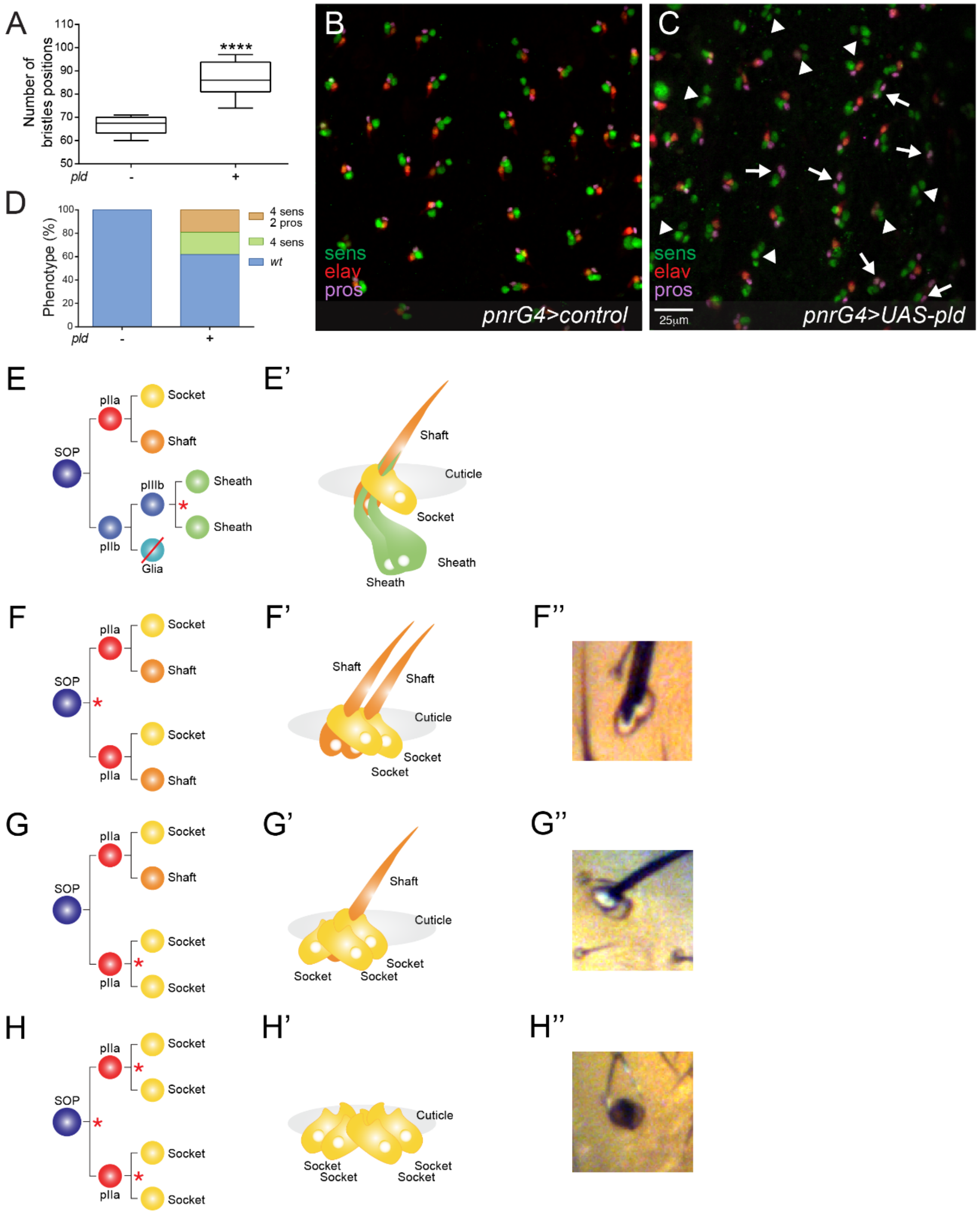

Medina-Yáñez et al., Figure S1.

Figure S1. PLD overexpression affects SO cell-fates. (A) Quantification of the number of Sensory Organ/Bristles **(B-D)** Confocal projection of the SOP cell lineage in the thorax. In green (senseless) marks the whole SOP lineage, in blue (elav) the neuron and in red (pros) the sheath. **(B)** Control. **(C)** PLD-GOF results in defects in binary cell-fate decisions. Arrow indicates a cluster with 2 sheath cells. The arrowhead indicates a cluster lacking neuron and sheath molecular markers. **(D)** Frequency of the 3 phenotypes **(E,**

F, G, H) Schematic representation of the abnormal differentiation that occurs during asymmetrical divisions of the SOP lineage. Note that different phenotypes have different levels of expressiveness, which is reflected as a different number of cells affected by overexpression of PLD, indicated by an asterisk. **(E', F', G', H')** Schematic representation of the correspondent acquired phenotype **(F'', G'', H'')** Higher magnification of macrochaetae with the correspondent acquired phenotype. Note that the frequency of SOP divisions was quantified considering the number of affected divisions. For a mechanoreceptor that presents 2sheath 2socket phenotype **(F`)**, only 1 cell division is affected **(F)**. However, in a mechanoreceptor with a 4so phenotype **(H')**, all 3 cell divisions showed a Notch gain of function **(H)**. The scale represents 5 µm **(B-C)**

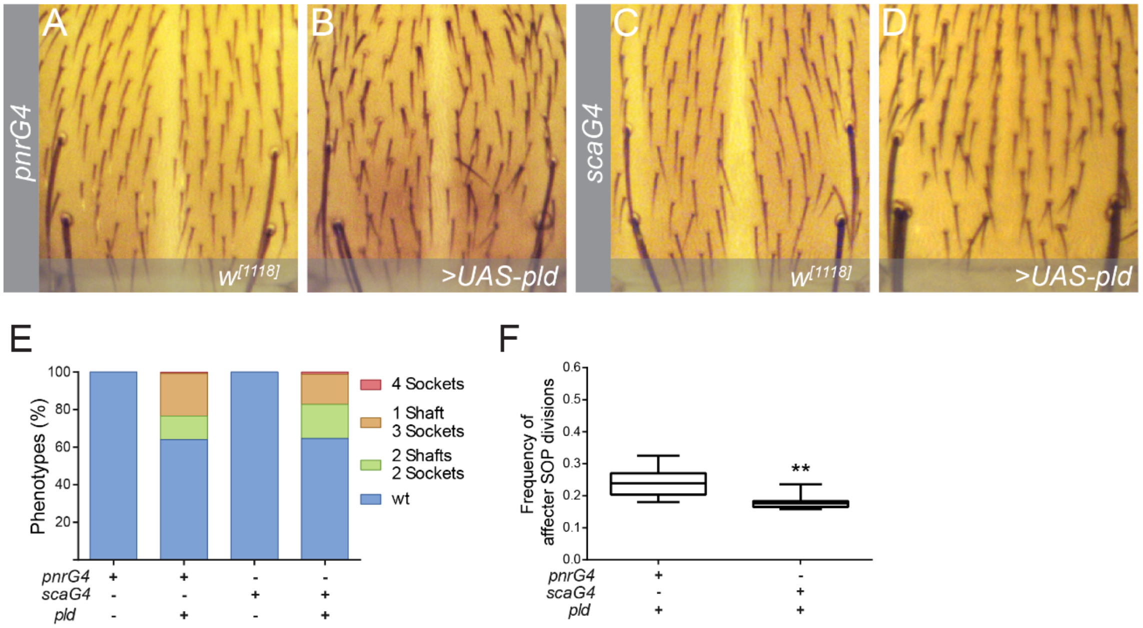

Medina-Yáñez et al., Figure S2.

Figure S2. Overexpression of PLD-PA using different Gal4 drivers. (A-D) Dorsal views of nota with a heterozygote background with two different drivers; **(A)** *pnrG4*, **(B)** *pnrG4>pld,* **(C)** *scaG4,* and **(D)** *scaG4>pld.* **(E)** Frequency of the 4 phenotypes for each case. **(F)** Quantification of the frequency of divisions affected by the gain function of Notch. (n = 10, ***p* <0.01)

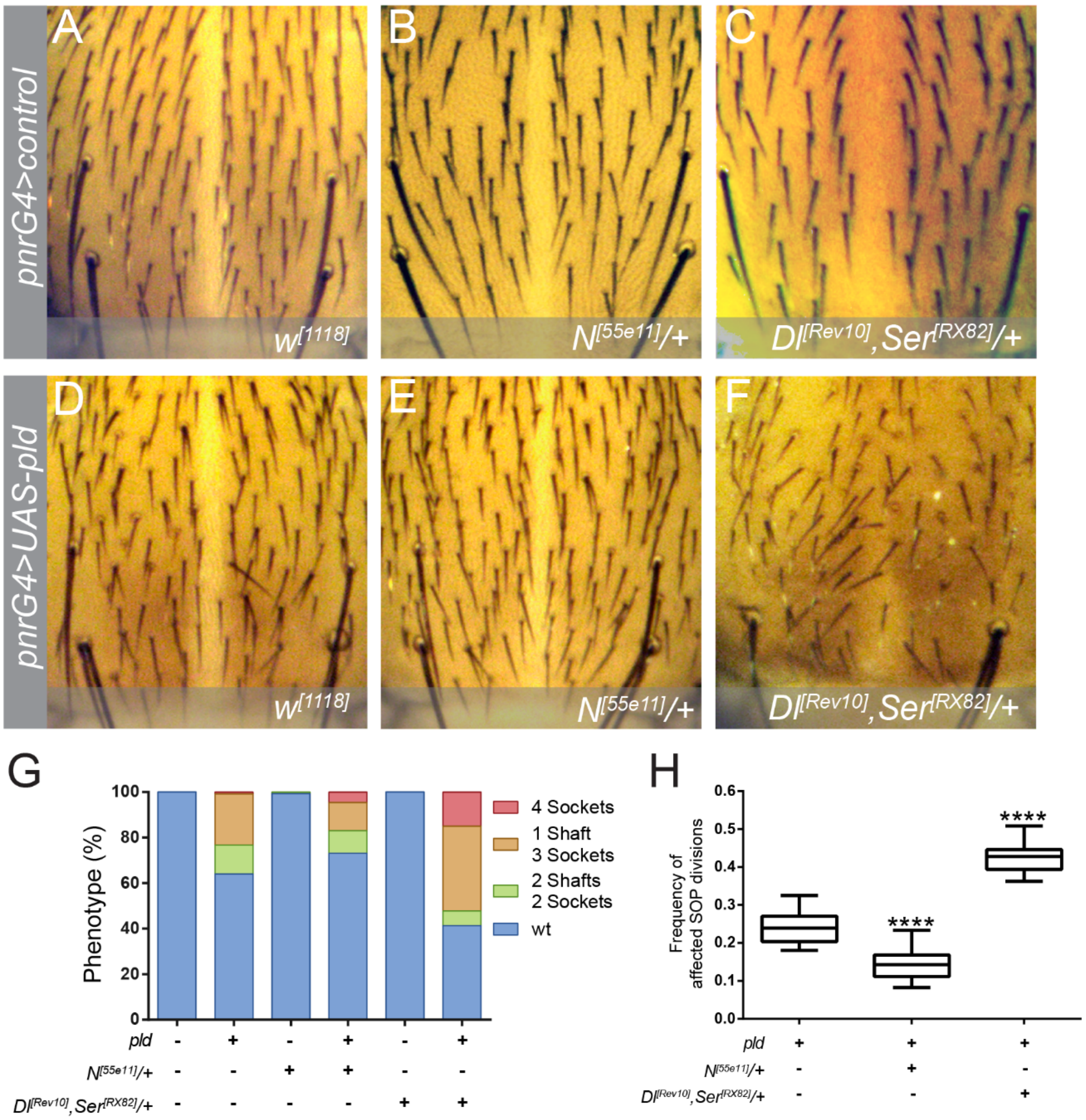

Medina-Yáñez et al., Figure S3.

Figure S3. Notch signaling receptors and ligands genetically modify the PLD overexpression phenotype. (A-F) Dorsal views of nota with a heterozygous background of the ligands and the receptor of Notch. Note that when analyzing the effect of null mutants on heterozygous alone **(B, C)**, no changes in SO's are observed, that is, they are haplosufficient for this trait. **(D-F)** We analyzed these mutants in the presence of PLD overexpression. **(G)** Frequency of the 4 phenotypes for genotype. **(H)** Quantification of the frequency of SOP divisions affected by the gain function of Notch. (n = 10, ****p <0.0001)

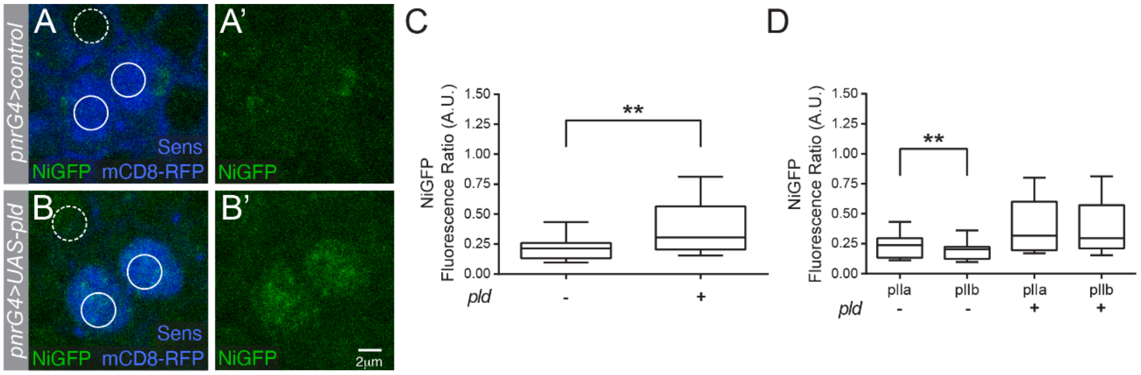

Medina-Yáñez et al., Figure S4.

Figure S4. PLD-PA increases Notch receptor signal. (A-O) Confocal projection of the SOP cell stained with senseless (red), the membrane with mCD8-RFP (red), and NiGFP (green). **(A-A')** Pupae control. **(B-B')** Pupae with overexpression of PLD **(C)** Quantification of fluorescence ratio in both conditions (n=10, $**p<0.01$). The scale represents 2 μm **(A-B')**.

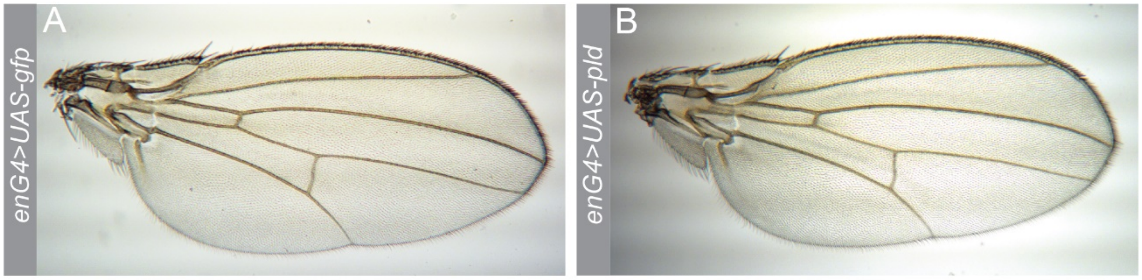

Medina-Yáñez et al., Figure S5.

Figure S5. Overexpression of PLD-PA in symmetric division context. (A-B) Horizontal views of left wings with a heterozygote background; **(A)** *enG4*, **(B)** *enG4>pld,* **(**n = 10)

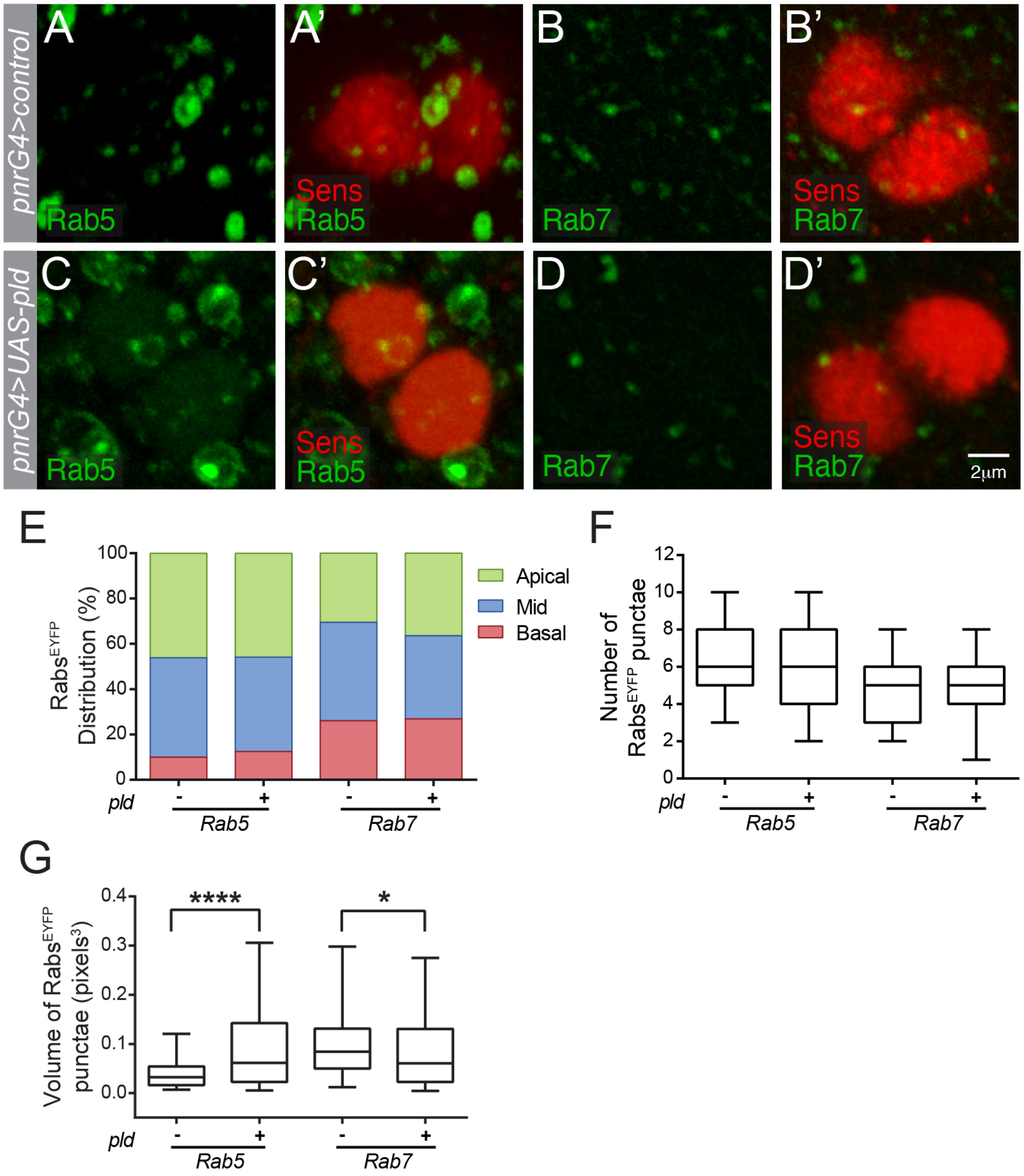

Medina-Yáñez et al., Figure S6.

Figure S6. PLD-PA promotes enlargement of the early endosome compartment and reduction of late endosome compartment (A-F') Confocal projection of the notum epithelium marked with Rabs fused with YFP (green) and senseless (red) to identify the SOP cells. **(A-B')** Control. **(C-D')** Overexpression of PLD. **(A-A', C-C')** $Rab5^{EYFP}$. **(B-B', D-D')** $Rab7^{EYFP.}$ Quantification of Rabs+ punctae **(F)** Spatial Distribution of Rabs+ punctae **(G)** Volume of Rabs+ punctae (A-A' n=24; B-B', C-C' n=27; D-D' n=13. * $p < 0.05$, **** $p < 0.0001$. The scale represents 2 μm (A-D')
